# The association between Resilience and psychological factors among patients suffering from cancer

**DOI:** 10.1192/j.eurpsy.2026.11558

**Published:** 2026-07-31

**Authors:** N. Bouattour, S. Omri, W. Ben kridiss, N. Smaoui, N. Charfi, R. Feki, I. Gassara, A. Khanfir, M. Mâalej, M. Mâalej, L. Zouari

**Affiliations:** 1 Psychiatry C, Hedi Chaker University Hospital; 2 Medical Carcinology, Habib Bourguiba University Hospital, Sfax, Tunisia

## Abstract

**Introduction:**

Cancer diagnosis and treatment often expose patients to significant psychological distress, including depression and anxiety, which can affect their overall well-being and treatment adherence. Resilience plays a crucial role in helping patients cope with these challenges.

**Objectives:**

The study aimed to assess the levels of resilience, depression, and anxiety in cancer patients and to explore the relationships among these variables.

**Methods:**

We conducted a descriptive and analytical cross-sectional study during January 2025, in the day care unit in the Medical Oncology department at the Habib Bourguiba University Hospital. Resilience was measured using the Connor-Davidson Resilience Scale (CD-RISC-10), while anxiety and depression were assessed with the Hospital Anxiety and Depression Scale (HAD).

**Results:**

Our study involved 72 patients. The average age was 54.6±11.5 years. Females represented 79.2% (n=57) with a sex ratio (M/F) equal to 0.017. The majority of our population was married (83.3%, n=60). Fifty-six patients (77.8%) had a high school educational level. Low socioeconomic status was reported in 65.3% (n=47). The most common cancer was breast cancer 65.3% (n=47). Six patients (8,6%) had a family history of similar cancer. Metastatic stage was observed in 55.5% of the patients (n=40). The prevalence of depression in our population was 52.77% (n=38), while anxiety prevalence was 56.94% (n=41). The average CD-RISC-10 score was 27.9±7,8. We found a statistically significant association between resilience and non-metastatic stage cancer (33.3±4.4 vs 23.65±7.1, p&lt;10 -3) and clinical improvement during treatment (19.98 vs 30.4, p&lt;10 -3 ). Resilience scores were significantly higher in non-depressed patients (34.11 ± 3.6 vs. 22.5 ± 6.2, p &lt; 10 -3 ) and in non-anxious patients (31.13 ± 3.6 vs. 25.4 ± 3.3, p = 0.009).

**Conclusions:**

Our findings highlight a high prevalence of depression and anxiety among cancer patients, with resilience significantly greater in those without metastatic disease and those showing clinical improvement during treatment. Higher resilience was also associated with lower levels of depression and anxiety. These results emphasize the importance of screening for psychological distress and fostering resilience to improve cancer patient’s; mental health and treatment outcomes.

**Disclosure of Interest:**

None Declared

